# Supplementary material for: Assembly of the infant gut microbiome and resistome are linked to bacterial strains in mother’s milk
Source: Nat Commun. 2025 Nov 22;16:11536. doi: 10.1038/s41467-025-66497-y (PMC12749357; doi:10.1038/s41467-025-66497-y)
Supplement: Supplementary file 2 — Description of Additional Supplementary Files [file 41467_2025_66497_MOESM2_ESM.pdf]

Description of Additional Supplementary Files

File name: Supplementary Data 1

Description: Cohort composition.

File name: Supplementary Data 2

Description: Samples metadata. Legend: SVD = Spontaneous Vaginal Delivery; AVD = Assisted Vaginal Delivery; CS = C-section; “Prenatal_abx” are defined as maternal antibiotic intake before the beginning of active labor.

File name: Supplementary Data 3

Description: Species-level taxonomic profiles as seen by MetaPhlAn4, using the *B. longum* subspecies marker genes provided by [37]

File name: Supplementary Data 4

Description: Strain sharing events, including strain sharing events between mother-infant pairs and strain persistence within the same infant over time. If a strain was shared between milk and infant stool at 1 month and also found in the infant at 6 months, it was counted as a single transmission event, assuming early transmission followed by persistence, to avoid over-counting.

File name: Supplementary Data 5

Description: HUMAnN3 functional profiles

File name: Supplementary Data 6

Description: DeepARG profiles
